# Factors influencing discharge readiness in acute leukemia patients and implications for nursing care: a mixed-methods study

**DOI:** 10.1590/1980-220X-REEUSP-2025-0456en

**Published:** 2026-11-09

**Authors:** Xin Tian, Xiumei Ma, Wei Liu, Jing Sun, Guoshan Gao, Shaochen Wang

**Affiliations:** 1 Shanxi Bethune Hospital, Department of Emergency Medicine, Taiyuan, China.; 2 Qiqihar Medical University, Nursing College, Qiqihar, China.; 3 The Second Affiliated Hospital of Qiqihar Medical University, Qiqihar, China.; 4 Ningxia Medical University, Yinchuan, China.

**Keywords:** Leukemia, Patient Discharge, Qualitative Research, Quantitative Research, Leucemia, Alta do Paciente, Pesquisa Qualitativa, Pesquisa Quantitativa

## Abstract

**Objective:**

This study aimed to investigate the level of discharge readiness among acute leukemia patients, as well as the factors that influence it.

**Method:**

A convergent mixed-methods design was employed. A quantitative survey was administered to 289 adult acute leukemia patients who were hospitalized at two tertiary-level Class A hospitals from July 2024 to July 2025. Additionally, semi-structured interviews were conducted with ten patients. Multiple linear regression was used to analyze the quantitative data and identify the factors influencing discharge readiness. The qualitative data were analyzed using thematic analysis based on Meleis’s transition theory.

**Results:**

The quantitative findings revealed that educational attainment, place of residence, method of medical payment, presence of chronic comorbidities, level of disease knowledge, unplanned readmission within 30 days, readiness for discharge, quality of discharge instructions, and social support significantly influenced patients’ discharge readiness (p < 0.05). Qualitative analysis further illuminated the roles of personal status, external support systems, nursing interventions, and role transitions in the discharge process. The mixed-methods findings demonstrated that the quantitative and qualitative results corroborated and complemented each other.

**Conclusion:**

Discharge readiness among acute leukemia patients was moderate and influenced by multiple factors. This study suggests the implementation of individualized discharge instructions and continuity-of-care models based on systematic assessments to provide evidence-based support for optimizing patient recovery and enhancing quality of life.

## RESUMO

**Objetivo:**

Este estudo teve como finalidade investigar o nível de prontidão para alta hospitalar entre pacientes com leucemia aguda e seus fatores influenciadores.

**Método:**

Foi adotado um desenho convergente de métodos mistos. Realizou-se uma pesquisa quantitativa com 289 pacientes adultos com leucemia aguda internados em dois hospitais terciários Classe A de julho de 2024 a julho de 2025; além disso, foram realizadas entrevistas semiestruturadas com 10 pacientes. Os dados quantitativos foram analisados por regressão linear múltipla para identificar os fatores que interferem na prontidão para alta, enquanto os dados qualitativos foram submetidos à análise temática baseada na teoria da transição de Meleis.

**Resultados:**

Os resultados quantitativos revelaram que escolaridade, local de residência, modalidade de pagamento médico, presença de comorbidades crônicas, nível de conhecimento sobre a doença, readmissão não planejada em 30 dias, estado geral de prontidão para alta, qualidade das orientações de alta e suporte social influenciam significativamente a prontidão dos pacientes para alta (*p*<0,05). A análise qualitativa aprofundou-se no papel do estado individual, sistemas de apoio externo, intervenções de enfermagem e transição de papéis no processo de desospitalização. Os resultados dos métodos mistos demonstraram que os dados quantitativos e qualitativos se corroboram e se complementam mutuamente.

**Conclusão:**

A prontidão para alta hospitalar dos pacientes com leucemia aguda encontra-se em nível moderado e é influenciada por múltiplos fatores. O estudo sugere a elaboração de orientações de alta individualizadas e modelos de cuidado continuado com base em avaliação sistemática, fornecendo evidências para otimizar a recuperação dos pacientes e melhorar sua qualidade de vida.

## INTRODUCTION

Acute leukemia (AL) can suppress normal hematopoietic function, leading to complications such as fatigue, bleeding, and infections^(1)^. According to the 2024 Global Cancer Statistics^(2)^, the number of new AL cases has increased to 487,000, and the increasing incidence rates pose a severe challenge to the global disease burden. Currently, multiagent combination chemotherapy is the primary treatment for AL and frequently induces adverse reactions, including bone marrow suppression, fatigue syndrome, and gastrointestinal discomfort^(3)^. These effects severely impair patients’ physiological functions and have multidimensional negative impacts on their psychological state, family support systems, and long-term quality of life^(4)^.

Meanwhile, the implementation of the national diagnosis-related group (DRG) system for medical insurance under the new healthcare reform often results in patients being discharged before fully recovering from bone marrow suppression. This leads to patients being discharged while still unfamiliar with daily self-care and inadequately prepared for discharge. Consequently, the frequency of readmissions and the rate of unplanned readmissions increase^(5)^. Against this backdrop, readiness for hospital discharge (RHD) has emerged as a critical indicator for assessing patients’ ability to transition safely from a hospital environment to a home or community care setting^(6)^. It encompasses not only physical and mental health status and disease awareness, but also multidimensional preparedness factors such as social support, psychological adaptation, and self-management capabilities. Numerous studies have demonstrated a significant correlation between readiness for hospital discharge and reduced 30-day readmission rates, improved treatment adherence, and enhanced long-term quality of life^(7)^. Some Western countries have established evidence-based guidelines for patient discharge readiness at the clinical practice level. Examples include the National Comprehensive Cancer Network (NCCN) in the United States, which recommends personalized transition care plans^(8)^, and the National Institute for Health and Care Excellence (NICE) in the United Kingdom, which emphasizes multidisciplinary collaboration and continuity of care models^(9)^. These systematic approaches integrate risk assessment, educational interventions, family involvement, and social resource linkages to significantly improve patient outcomes. In contrast, China is still in the early exploratory phase in this field. Currently, there is a lack of localized risk assessment tools and structured intervention protocols for AL patients. Discharge processes often prioritize routine health education while neglecting individualized attention. Notably, AL patients exhibit high heterogeneity in discharge readiness due to treatment complexity, prolonged rehabilitation cycles, and variable complications. Single research methodologies struggle to capture these intricate causes and mechanisms comprehensively. Mixed-methods research designs, which integrate quantitative and qualitative data, are particularly well-suited to exploring such complex factors. Previous studies on chronic diseases^(10)^ and oncological rehabilitation^(11)^ have demonstrated the efficacy of this approach in identifying influencing factors and elucidating underlying mechanisms. However, the application of mixed methods to AL patients is unexplored, and there is a lack of systematic evidence to support clinical decision-making.

Therefore, this study employs a convergent mixed-methods design based on Meleis’s transitional theory framework^(12)^. The study aims to comprehensively assess the current state and influencing factors of discharge readiness among AL patients. Cross-validation and complementary integration of qualitative and quantitative data will reveal the mechanisms influencing the discharge transition. The findings are expected to provide evidence-based support for establishing an AL patient discharge readiness assessment and intervention system tailored to China’s healthcare and cultural contexts. These findings will provide a foundation for developing a system to assess and improve the discharge preparation of AL patients. This will improve patients’ quality of life, reduce the burden on family caregivers, and optimize the allocation of healthcare resources. The ultimate goal is to advance the field of hematologic oncology nursing.

## METHOD

### Study Design and Setting

This study uses a convergent mixed-methods design, collecting quantitative and qualitative data simultaneously. The data are analyzed separately before being integrated to gain a more comprehensive and in-depth understanding of the factors influencing discharge readiness among AL patients. The quantitative component uses an online questionnaire to assess discharge readiness levels and gather additional information. The qualitative component uses a semi-structured, descriptive approach to explore patients’ experiences with discharge readiness.

### Population and Sample

We selected patients with acute lymphoblastic leukemia (AL) from the hematology departments of two grade A, level 3 hospitals — one in Qiqihar City, Heilongjiang Province, China, and the other in Yinchuan City, Ningxia Hui Autonomous Region, China — as the study subjects using convenience sampling between July 2024 and July 2025. The inclusion criteria were as follows: 1) age ≥18 years, 2) a diagnosis that meets the latest World Health Organization (WHO) criteria for acute leukemia, confirmed by bone marrow aspiration, and 3) an understanding of and voluntary participation in the study. The exclusion criteria were as follows: (1) a history of other malignancies or severe impairment of vital organs (e.g., the liver, kidneys, or lungs); (2) cognitive or communication impairments that prevent completion of the questionnaire; and (3) a history of psychiatric disorders.

For quantitative research, the sample size should be at least five to ten times the number of study variables^(13)^. This study included 24 independent variables. Considering factors such as invalid responses to the questionnaire, the sample size for the survey was increased by 20%, resulting in a range of 150–300 cases. This study included a total of 289 cases. In this qualitative study, purposive sampling with maximum variation was employed to recruit AL patients as research subjects representing diverse genders, ages, educational backgrounds, and other characteristics. Semi-structured interviews were conducted. The sample size was determined according to the principle of information saturation. Data collection ceased after conducting two additional interviews without eliciting new themes. Ultimately, the analysis included 10 patients.

### Measures

#### Socio-Demographic Characteristics

The researcher designed the survey questionnaire on the basis of prior studies and theoretical frameworks. It requested demographic information, such as gender, age, and educational attainment, as well as disease-related details, such as duration of illness, length of hospitalization, and presence of chronic conditions.

#### Readiness for Hospital Discharge Scale (RHDS)

This study used the scale adapted into Chinese by Lin et al.^(14)^ in 2014. The scale has three dimensions and 12 items: Personal State (Items 1–3), Adaptive Capacity (Items 4–8), and Anticipatory Support (Items 9–12). The total score ranges from 0 to 120, with each item scored from 0 to 10. Scores of 9–10 indicate a very high level of preparedness, 8–8.9 a high level, 7–7.9 a moderate level, and 0–7 a low level. A higher total score indicates greater preparedness for discharge. The Cronbach’s α coefficient for this scale is 0.890. In this study, it is 0.893.

#### Quality of Discharge Teaching Scale (QDTS)

The Chinese version of the Discharge Teaching Quality Scale was revised by Chinese scholar Wang et al.^(15)^. The scale, developed by Weiss and Lokken^(16)^, comprises three dimensions: content that should be mastered before discharge, content that was actually mastered before discharge, and the effectiveness of discharge teaching skills. The scale consists of 24 items in total. The scale measures the quality of discharge instructions by calculating the total scores of the “actual knowledge acquired before discharge” and “skills and effectiveness of discharge instructions” dimensions. Higher total scores indicate better instruction quality. The Cronbach’s α coefficient for this scale is 0.920. In this study, it is 0.939.

#### Social Support Rating Scale (SSRS)

Xiao^(17)^ developed this 10-item scale in 1987. It comprises three dimensions: objective support, subjective support, and utilization of support. The scoring criteria are as follows: Items 1–4 and 8–10 allow only one selection per item and are scored from 1 to 4 points, respectively. Item 5 is subdivided into four categories (A, B, C, and D), each of which is scored from 1 to 4 points, ranging from “none” to “full support.” Items 6 and 7 are scored as follows: 0 points if “no sources” is selected and the number of sources listed under “the following sources” is counted as points. The total SSRS score is the sum of the scores across all ten items. The Cronbach’s α coefficient for this scale is 0.892. In this study, it is 0.838.

#### Hospital Anxiety and Depression Scale (HADS)

It was developed by Zigmond and Snaith^(18)^ in 1983. This scale screens for potential symptoms of anxiety and depression in hospitalized patients. It has two dimensions, anxiety and depression, and a total of 14 items. Scores of 0–7 on either dimension indicate the absence of symptoms. Scores of 8–10 suggest the presence of possible symptoms, while scores of 11–21 confirm it. The Cronbach’s α coefficient for this scale is 0.875. In this study, it is 0.927.

#### Interview Outline

Based on a literature review and team discussions, the researcher developed a preliminary interview outline focused on factors that promote or hinder discharge readiness among AL patients. The outline was guided by transition theory. Six hematology-oncology experts reviewed the outline, and two eligible AL patients participated in pilot interviews to finalize the framework.

#### Data Collection

The head nurse or the patient’s assigned nurse conducted quantitative data collection. They introduced the purpose and significance of the study to patients and obtained their consent prior to data collection. Researchers conducted one-on-one on-site interviews using standardized prompts. For qualitative data, researchers trained in qualitative interviewing techniques conducted fully recorded interviews, focusing on documenting patients’ nonverbal cues, such as facial expressions and gestures. Within 24 hours after the interview, the first and second authors transcribed the audio recordings and handwritten notes into text. Any unclear content was clarified via telephone follow-up with patients.

#### Data Analysis

SPSS 26.0 software was used for quantitative data analysis. Intergroup comparisons used t-tests or one-way ANOVA, and correlations were assessed using Pearson’s method. Multivariate linear regression analysis was conducted with the RHDS total score as the dependent variable. To ensure robustness, the models underwent collinearity (variance inflation factor (VIF)), residual independence (Durbin–Watson), and normality tests. Qualitative data analysis was conducted using Colaizzi’s sevenstep method^(19)^. Two researchers coded and crosschecked the data independently, and discrepancies were resolved through group discussion. Member checking was employed to ensure the reliability of the results.

### Ethical Considerations

This study was approved by the hospital ethics committee (approval no. 2024-02-005-01). All participants provided informed consent and voluntarily enrolled in the study. Trained researchers conducted qualitative data collection. The interviews were audio-recorded and transcribed. Member checking and inter-rater reliability checks ensured the credibility and consistency of the data. The study was conducted in accordance with the Declaration of Helsinki.

## RESULTS

### Sample Characteristics

After the data was collected, two researchers independently reviewed and verified the questionnaires. Based on predefined quality control criteria, such as the presence of obvious logical errors or patterned responses, two invalid questionnaires were excluded. Ultimately, the analysis included 289 valid questionnaires, yielding a response rate of 99.3%. Most patients were female (58.1%), aged 60 years or older (44.3%), had an educational attainment level of junior high school or lower (61.9%), and were from rural areas (48.1%). The patients’ general characteristics are summarized in Table 1.

**Table 1:** The results of the univariate analysis of the general information and readiness for discharge of the study participants (n = 289) – Heilongjiang, China, 2025.

Variables	Number of participants (percentage)	Readiness for discharge (score, x̄ ± S	t/F	P-value
Gender			3.753^1)^	<0.001
Male	121(41.9)	97.21 ± 14.57		
Female	168(58.1)	89.43 ± 19.15		
Age			5.238^2)^	0.006
<45	68(23.5)	97.38 ± 17.21		
45–60	93(32.2)	94.03 ± 18.92		
≥60	128(44.3)	89.21 ± 16.59		
Educational Attainment			17.542^2)^	<0.001
Junior high school or below	179(61.9)	88.11 ± 17.83		
High school or college	93(32.2)	99.77 ± 15.08		
Bachelor’s degree or above	17(5.9)	102.12 ± 14.89		
Marital Status			4.253^1)^	<0.001
Married	227(78.5)	94.94 ± 16.24		
Unmarried/Divorced/Widowed	62(21.5)	84.42 ± 20.62		
Place of Residence			20.385^2)^	<0.001
Rural	139(48.1)	86.27 ± 18.18		
Town	43(14.9)	101.00 ± 14.55		
Urban	107(37.0)	97.67 ± 15.40		
Living with family			2.676^1)^	0.008
Yes	273(94.5)	93.36 ± 17.62		
No	16(5.5)	81.25 ± 17.03		
Medical Payment Method			10.786^2)^	<0.001
New Rural Cooperative Medical Scheme	126(43.6)	86.30 ± 17.58		
Urban Resident Medical Insurance	83(28.7)	97.80 ± 16.86		
Employee Medical Insurance	74(25.6)	97.03 ± 16.22		
Other	6(2.1)	102.50 ± 10.46		
Monthly per capita household income			36.022^2)^	<0.001
<3000	162(56.1)	86.10 ± 18.89		
3000–5000	95(32.9)	98.61 ± 11.28		
>5000	32(11.1)	108.44 ± 10.23		
Primary caregiver			10.298^2)^	<0.001
Parents	27(9.3)	100.04 ± 17.35		
Spouse	141(48.8)	96.81 ± 14.18		
Children	100(34.6)	86.39 ± 20.27		
Other	21(7.3)	85.52 ± 16.48		
Working Status			13.152^2)^	<0.001
Unemployed	177(61.2)	89.52 ± 18.47		
Resigned/Retired	85(29.4)	94.73 ± 15.43		
Active	27(9.3)	107.00 ± 11.27		
Duration of illness			16.829^2)^	<0.001
<1 month	29(10.0)	80.21 ± 16.96		
1–6 months	49(17.0)	85.39 ± 13.79		
>6 months	211(73.0)	96.09 ± 17.49		
Chronic Disease Comorbidity			–6.601^1)^	<0.001
Yes	130(45.0)	85.56 ± 18.73		
No	159(55.0)	98.51 ± 14.61		
Length of Hospital Stay			2.373^2)^	0.070
<7 days	71(24.6)	92.89 ± 19.02		
7–14 days	113(39.1)	94.03 ± 16.83		
14–21 days	58(20.1)	87.43 ± 15.02		
≥21 days	47(16.3)	95.64 ± 20.23		
Disease Awareness Level			55.303^2)^	<0.001
Very familiar	163(56.4)	101.56 ± 13.58		
Somewhat familiar	72(24.9)	85.69 ± 15.24		
Not very familiar	40(13.8)	77.28 ± 15.88		
Not at all familiar	14(4.8)	69.36 ± 9.94		
Unplanned readmission within 30 days			–6.262^1)^	<0.001
Yes	35(12.1)	76.11 ± 16.78		
No	254(87.9)	94.97 ± 16.69		
Readiness for discharge			10.036^1)^	<0.001
Yes	249(86.2)	96.31 ± 15.12		
No	40(13.8)	70.13 ± 16.50		

Note: *t* = independent sample *t* test; *F* = one-way ANOVA;

^1)^ = *t* value;

^2)^ = *F* value.

### The RHDS Scores of AL Patients

The RHDS score for AL patients was 92.69 ± 17.77, with subscores of 21.96 ± 6.13 for personal status, 38.09 ± 10.04 for adaptive capacity, and 32.63 ± 6.83 for anticipated support. The mean score for all items was 7.72 ± 1.48, indicating a moderate level of discharge readiness.

### Univariate Analysis (Table 1 and Table 2)

**Table 2:** Correlation analysis of discharge readiness among study participants (N = 289) – Heilongjiang, China, 2025.

Variable	Readiness for discharge	Quality of discharge instructions	Social support	Anxiety and depression
Readiness for Discharge	1			
Quality of Discharge Instructions	0.737**	1		
Social Support	0.570**	0.542**	1	
Anxiety and Depression	–0.597**	–0.695**	–0.502**	1

Note: **P < 0.01.

### Multivariate Linear Regression Analysis

A multivariate linear regression analysis was conducted with the RHDS total score as the dependent variable and the statistically significant variables from the univariate analysis as the independent variables. The results indicate that educational attainment, place of residence, method of medical payment, presence of comorbid chronic diseases, level of disease knowledge, occurrence of unplanned readmission within 30 days, readiness for discharge, quality of discharge instructions, and social support influence discharge readiness among AL patients. These factors account for 71.7% of the total variance (Table 3).

**Table 3:** Multivariate linear regression analysis of discharge readiness among study participants (N = 289) – Heilongjiang, China, 2025.

Variables	B	SE	β	t	P
Constant	29.999	11.679		2.569	0.011
Educational Attainment	3.173	1.280	0.108	2.479	0.014
Residence = City	8.395	2.297	0.168	3.655	<0.001
Residence = Town	7.378	2.802	0.201	2.634	0.009
Medical Payment Method = Urban Resident Medical Insurance	5.929	2.044	0.151	2.901	0.004
Medical Payment Method = Employee Medical Insurance	13.729	2.909	0.338	4.719	<0.001
Chronic Disease Comorbidity	4.093	1.300	0.115	3.149	0.002
Level of disease awareness	–2.171	1.021	–0.109	–2.128	0.034
Whether there was an unplanned readmission within 30 days	6.693	1.893	0.123	3.536	<0.001
Readiness for discharge	–7.172	1.988	–0.140	–3.607	<0.001
Quality of Discharge Instructions	0.310	0.046	0.369	6.682	<0.001
Social Support	0.257	0.096	0.130	2.661	0.008

Note: R_2_ = 0.742, adjusted R2 = 0.717; F = 30.191, *P* < 0.001.

### Qualitative Research Findings

#### Theme 1: Impact of Personal Circumstances on Readiness for Discharge

A patient’s readiness for discharge is closely linked to their physical functioning, psychological coping skills, and understanding of their disease. Due to the side effects of chemotherapy, most patients experience reduced physical strength and symptom distress, which further affects their psychological state. Some patients gradually develop positive cognitive patterns during prolonged illness, thereby enhancing their ability to cope.

*“After chemotherapy, I feel extremely fatigued. At home, I can only rest; any activity worsens the discomfort” (P3).*

*“Despite the financial strain, I have gradually come to terms with it, seeing it as a sacrifice for a better future.” (P2).*

#### Theme 2: Role of External Support Systems

Family support plays a pivotal role in a patient’s recovery by alleviating anxiety and increasing confidence. Meanwhile, economic assistance and emotional encouragement from society and the government can significantly reduce patients’ burdens.

*“My family cares for me every day, making me feel incredibly secure.” (P6).*

*“The government provided me with low-income assistance, which eased my financial burden considerably.” (P8).*

#### Theme 3: Supportive Role of Nursing Interventions

Standardized discharge instructions and continuity of care services improve patients’ ability to manage their disease and provide ongoing support for home-based care. Patients consistently reported that guidance from healthcare providers bolstered their confidence in self-management.

*“The nurse explained in detail how to manage my care at home, which gave me greater peace of mind.” (P4).*

*“After discharge, doctors and nurses followed up via WeChat, promptly addressing any questions I had.” (P2).*

#### Theme 4: Role Perception and Transformation

During the disease-to-health transition, patients transitioned from anxiety and helplessness to gradual acceptance and adaptation. A positive role perception improved discharge readiness and social reintegration.

*“At first, I felt restricted by my illness. Now that I’ve been discharged, I feel no different from anyone else” (P5).*

#### Mixed-Methods Findings

Framed by transition theory, this study revealed that the readiness of AL patients for discharge exhibits complementarity at the transition condition level and extensibility at the levels of nursing intervention and transition nature.

## DISCUSSION

### Focus on Patients’ Individual Circumstances, Understand Their Needs, and Guide Them Toward Proactive Coping Strategies

In terms of individual status, quantitative and qualitative research complement each other. Quantitative research, supported by data, identified educational and disease knowledge levels as two key variables. These findings are validated across diseases^(20)^. Qualitative research delves into the underlying causes of individual status, uncovering the concept of self-care competence and supplementing the quantitative findings. The results suggest that patients with strong self-care capabilities pay close attention to infection control details and can adjust based on their circumstances. This self-care capability establishes a clear framework prior to discharge. In contrast, patients with weaker self-care capabilities exhibited confusion and anxiety. One patient mentioned this in an interview: “I don’t know what to do once I get home.” Their lack of proper self-care planning diminished the effectiveness of discharge preparation. This finding is consistent with the quantitative results, which suggest that individuals with higher educational attainment typically have stronger self-care learning abilities. According to the Knowledge-Belief-Behavior (KBB) theory, the greater one’s understanding of disease treatment, the stronger their motivation for self-care^(21)^. The two studies complement each other. The quantitative study established a framework for individual status and identified factors that influence AL patients’ readiness for discharge. The qualitative study explained the underlying rationale and mechanisms through which these characteristics exert their influence. Therefore, it is recommended that healthcare providers proactively assess patients’ physical and mental conditions, as well as their self-care capabilities. They should actively encourage patients to transform passive medical compliance into active participation in rehabilitation. At the same time, they should develop visual, interactive educational tools tailored to patients with low levels of education to reduce the complexity of health education and effectively improve discharge readiness.

### Building and Improving Patient Support Systems

Quantitative research indicates that patients’ social support influences their readiness for discharge. This finding is consistent with the theme of “external support systems” identified in qualitative research. Patient interview statements such as “My spouse quit their job to provide full-time care” and “Comprehensive care from family members” corroborate emotional comfort and family care, both of which are forms of subjective support. Conversely, examples such as “funding from Waterdrop” and “reimbursement through government subsistence allowances” demonstrate the practical role of material assistance within objective support. Furthermore, quantitative research revealed that patients residing in rural areas are less ready for discharge than those living in urban areas. In-depth analysis suggests that urban patients can access medical treatment more quickly in emergencies. This results in lower anxiety and a greater sense of security, enhancing their readiness for discharge^(22)^. Therefore, it is recommended that healthcare professionals recognize the important role of caregivers and encourage family members to play an active role in caring for patients. This will provide patients with more support. At the same time, collaborative efforts between hospitals, communities, and families should be strengthened. Continuity of care centers should be established in partnership with primary healthcare institutions to conduct regular follow-ups and provide telemedicine consultations. Social support networks should be improved, and the integration of medical insurance and charitable programs should be promoted. In particular, a targeted assistance system should be established to address the medical needs and alleviate the financial burdens of rural populations.

### Enhancing Nurse‒Patient Communication and Improving the Continuity of Care Services

The results suggest that the quality of discharge instructions influences readiness for discharge, which is consistent with previous studies’ findings^(23,24)^. Qualitative research also indicates that continuity of care services can improve patients’ readiness for discharge. Analysis indicates that this may be because patients with acute lymphoblastic leukemia (AL) are not fully recovered by the time they are discharged. Treatment-related toxic reactions coexist with long-term self-management needs, resulting in a greater demand for health guidance than in the general patient population. Accurate and timely continuity of care services can effectively boost patients’ confidence in returning home and their ability to manage their own care. This reduces the incidence of complications and improves readiness for discharge. Therefore, it is recommended that healthcare professionals optimize discharge preparation guidance and engage patients through various educational initiatives. At the same time, the quality of continuity of care services should be improved. Hospitals could establish platforms to provide continuous care services via regular telephone calls, WeChat, and “Internet + Nursing” methods^(25)^. These measures would address patients’ concerns after leaving the hospital and provide comprehensive, full-cycle rehabilitation support.

### Focus on Disease Transition and Social Role Reconstruction

The study findings indicate that patients with chronic diseases or unplanned readmissions are less prepared for discharge, which aligns with Yang’s et al. findings^(26)^. These patients require more complex treatment and care plans, which can lead to a significant increase in complications and unplanned readmissions. Qualitative analysis revealed that role perception and transition are key themes influencing readiness for discharge. From a transition theory perspective, AL patients experience an environmental transition during the discharge phase, as well as a process of identity reconstruction — specifically, a shift from being dependent hospital patients to becoming primary caregivers who are responsible for self-management at home. This process of identity transition involves adjustments to the self-concept. According to self-regulation theory^(27)^, when individuals are faced with a significant health threat, they develop relevant behavioral strategies based on their understanding of the disease. Patients who perceive their illness as a manageable chronic condition are more likely to adopt proactive coping behaviors, thereby improving their readiness for discharge. Meanwhile, illness identity theory suggests that patients with chronic diseases must integrate their illness identity into their self-concept^(28)^. In this study, some patients demonstrated active identity integration. For example, they said, “I’ll be fully recovered once I’m discharged,” reflecting their reconstruction of their illness identity. Therefore, preparing for discharge involves more than acquiring knowledge; it also involves reconstructing one’s identity and undergoing a psychological transition. Future interventions should expand beyond basic health education to include role empowerment and identity integration to enhance patients’ self-management capacity.

## CONCLUSION

This study used a convergent mixed-methods design and found that patients with acute leukemia have moderate levels of discharge readiness. This finding confirms that readiness is influenced by a multidimensional interplay of factors, including personal characteristics, external support, nursing services, and perceptions of health roles. However, existing nursing models demonstrate shortcomings in long-term management from hospital to home. These models prioritize symptom control while neglecting the sustainable development of psychosocial adaptation and self-management capabilities.

The study had several limitations. First, the sample was restricted to two Grade A tertiary hospitals in northern China, which may limit the generalizability of the results. Second, there were methodological challenges in providing health education to low-literacy patients. Third, continuity of care services are limited in rural areas, and structural deficiencies may impact long-term rehabilitation outcomes. Future research may focus on the following: 1. Conducting multicenter, large-sample, longitudinal studies to dynamically track changes in discharge readiness and its influencing factors, as well as to identify critical intervention windows. 2. Developing and validating visual, interactive health education tools to increase disease awareness and self-management capabilities among patients with low literacy levels. 3. Establishing a digital continuity of care model based on “hospital–community–home” collaboration. This model should pay particular attention to service accessibility for rural patients, optimize nursing resource allocation, and improve long-term quality of life. Integrating research findings with theoretical frameworks provides actionable strategies for discharge readiness interventions, emphasizing the pivotal role of multidimensional interactive mechanisms in enhancing acute leukemia patients’ rehabilitation outcomes and quality of life.

## Data Availability

The entire dataset supporting the results of this study is available upon request to the corresponding author.
