# Supplementary material for: Factors influencing discharge readiness in acute leukemia patients and implications for nursing care: a mixed-methods study
Source: Rev Esc Enferm USP. 2026 Nov 9;60:e20250456. doi: 10.1590/1980-220X-REEUSP-2025-0456en (PMC13747296; doi:10.1590/1980-220X-REEUSP-2025-0456en)
Supplement: Translation

# Translation: Factores que influyen en la preparación para el alta en pacientes con leucemia aguda y sus implicaciones para la atención de enfermería: un estudio de métodos mixtos

**DOI:** 10.1590/1980-220X-REEUSP-2025-0456es

**Authors:** Xin Tian, Xiumei Ma, Wei Liu, Jing Sun, Guoshan Gao, Shaochen Wang

**Affiliations:** 1 Shanxi Bethune Hospital, Department of Emergency Medicine, Taiyuan, China.; 2 Qiqihar Medical University, Nursing College, Qiqihar, China.; 3 The Second Affiliated Hospital of Qiqihar Medical University, Qiqihar, China.; 4 Ningxia Medical University, Yinchuan, China.

**Keywords:** Leucemia, Alta del Paciente, Investigación Cualitativa, Investigación Cuantitativa

## Abstract

**Objetivo:**

El objetivo de este estudio fue investigar el nivel de preparación para el alta entre los pacientes con leucemia aguda, así como los factores que influyen en él.

**Método:**

Se empleó un diseño convergente de métodos mixtos. Se realizó una encuesta cuantitativa a 289 pacientes adultos con leucemia aguda que estuvieron hospitalizados en dos hospitales terciarios de Clase A entre julio de 2024 y julio de 2025. Además, se realizaron entrevistas semiestructuradas a diez pacientes. Se utilizó la regresión lineal múltiple para analizar los datos cuantitativos e identificar los factores que influyen en el nivel de preparación para el alta. Los datos cualitativos se analizaron mediante un análisis temático basado en la teoría de la transición de Meleis.

**Resultados:**

Los hallazgos cuantitativos revelaron que el nivel educativo, el lugar de residencia, la forma de pago de los servicios médicos, la presencia de comorbilidades crónicas, el nivel de conocimiento sobre la enfermedad, el reingreso no planificado en un plazo de 30 días, la preparación para el alta, la calidad de las instrucciones de alta y el apoyo social influyeron significativamente en la preparación para el alta de los pacientes (p < 0,05). El análisis cualitativo esclareció aún más el papel que desempeñan la situación personal, los sistemas de apoyo externos, las intervenciones de enfermería y las transiciones de roles en el proceso de alta. Los hallazgos de los métodos mixtos demostraron que los resultados cuantitativos y cualitativos se corroboraban y complementaban entre sí.

**Conclusión:**

La preparación para el alta entre los pacientes con leucemia aguda fue moderada y estuvo influenciada por múltiples factores. Este estudio sugiere la implementación de instrucciones de alta individualizadas y modelos de continuidad de la atención basados en evaluaciones sistemáticas para brindar apoyo basado en la evidencia con el fin de optimizar la recuperación del paciente y mejorar su calidad de vida.

## INTRODUCCIÓN

La leucemia aguda (LA) puede suprimir la función hematopoyética normal, lo que conduce a complicaciones como fatiga, hemorragias e infecciones^(1)^. Según las Estadísticas Globales sobre el Cáncer de 2024^(2)^, el número de nuevos casos de LA ha aumentado a 487 000, y las tasas de incidencia en aumento representan un grave desafío para la carga mundial de la enfermedad. Actualmente, la quimioterapia combinada con múltiples agentes es el tratamiento principal para la LA y con frecuencia provoca reacciones adversas, entre ellas la supresión de la médula ósea, el síndrome de fatiga y molestias gastrointestinales^(3)^. Estos efectos afectan gravemente las funciones fisiológicas de los pacientes y tienen repercusiones negativas multidimensionales en su estado psicológico, sus sistemas de apoyo familiar y su calidad de vida a largo plazo^(4)^.

Mientras tanto, la implementación del sistema nacional de grupos relacionados con el diagnóstico (DRG) para el seguro médico, en el marco de la nueva reforma de salud, a menudo da lugar a que los pacientes sean dados de alta antes de recuperarse por completo de la supresión de la médula ósea. Esto hace que los pacientes sean dados de alta sin estar aún familiarizados con el autocuidado diario y sin estar adecuadamente preparados para ello. En consecuencia, aumentan la frecuencia de las readmisiones y la tasa de readmisiones no planificadas^(5)^. En este contexto, la preparación para el alta hospitalaria (readiness for hospital discharge - RHD) se ha convertido en un indicador fundamental para evaluar la capacidad de los pacientes de realizar una transición segura del entorno hospitalario al hogar o a un entorno de atención comunitaria^(6)^. Abarca no solo el estado de salud física y mental y la conciencia de la enfermedad, sino también factores de preparación multidimensionales como el apoyo social, la adaptación psicológica y las capacidades de autocontrol. Numerosos estudios han demostrado una correlación significativa entre la preparación para la alta hospitalaria y la reducción de las tasas de reingresos a los 30 días, una mejor adherencia al tratamiento y una mayor calidad de vida a largo plazo^(7)^. Algunos países occidentales han establecido guías basadas en la evidencia para la preparación de los pacientes para la alta a nivel de la práctica clínica. Entre los ejemplos se incluyen la Red Nacional Integral del Cáncer (National Comprehensive Cancer Network - NCCN) de Estados Unidos, que recomienda planes personalizados de atención de transición^(8)^, y el Instituto Nacional para la Salud y la Excelencia Clínica (National Institute for Health and Care Excellence - NICE) del Reino Unido, que hace hincapié en la colaboración multidisciplinaria y los modelos de continuidad de la atención^(9)^. Estos enfoques sistemáticos integran la evaluación de riesgos, las intervenciones educativas, la participación familiar y los vínculos con recursos sociales para mejorar significativamente los resultados de los pacientes. En contraste, China aún se encuentra en una fase exploratoria temprana en este campo. Actualmente, hay una falta de herramientas de evaluación de riesgo adaptadas al contexto local y de protocolos de intervención estructurados para pacientes con LA. Los procesos de alta suelen dar prioridad a la educación en salud de rutina, mientras que descuidan la atención individualizada. Cabe destacar que los pacientes con LA presentan una alta heterogeneidad en cuanto a su preparación para el alta debido a la complejidad del tratamiento, los ciclos prolongados de rehabilitación y las complicaciones variables. Las metodologías de investigación únicas tienen dificultades para captar de manera integral estas causas y mecanismos intrincados. Los diseños de investigación de métodos mixtos, que integran datos cuantitativos y cualitativos, son particularmente adecuados para explorar factores tan complejos. Estudios previos sobre enfermedades crónicas^(10)^ y rehabilitación oncológica^(11)^ han demostrado la eficacia de este enfoque para identificar factores influyentes y dilucidar los mecanismos subyacentes. Sin embargo, la aplicación de métodos mixtos a pacientes con LA no se ha explorado, y hay una falta de evidencia sistemática que respalde la toma de decisiones clínicas.

Por lo tanto, este estudio emplea un diseño convergente de métodos mixtos basado en el marco de la teoría de la transición de Meleis^(12)^. El estudio tiene como objetivo evaluar de manera integral el estado actual y los factores que influyen en la preparación para el alta entre los pacientes con LA. La validación cruzada y la integración complementaria de datos cualitativos y cuantitativos revelarán los mecanismos que influyen en la transición hacia el alta. Se espera que los hallazgos brinden un respaldo basado en la evidencia para establecer un sistema de evaluación e intervención de la preparación para el alta de pacientes con LA adaptado a los contextos sanitarios y culturales de China. Estos hallazgos sentarán las bases para desarrollar un sistema que evalúe y mejore la preparación para el alta de los pacientes con LA. Esto mejorará la calidad de vida de los pacientes, reducirá la carga sobre los cuidadores familiares y optimizará la asignación de los recursos de salud. El objetivo final es avanzar en el campo de la enfermería en oncología hematológica.

## MÉTODO

### Diseño y Entorno Del Estudio

Este estudio utiliza un diseño convergente de métodos mixtos, que recolecta datos cuantitativos y cualitativos simultáneamente. Los datos se analizan por separado antes de integrarlos para obtener una comprensión más integral y profunda de los factores que influyen en la preparación para el alta entre los pacientes con LA. El componente cuantitativo utiliza un cuestionario en línea para evaluar los niveles de preparación para el alta y recopilar información adicional. El componente cualitativo utiliza un enfoque descriptivo y semiestructurado para explorar las experiencias de los pacientes con respecto a la preparación para el alta.

### Población y Muestra

Seleccionamos a pacientes con leucemia linfoblástica aguda (LA) de los departamentos de hematología de dos hospitales de grado A y nivel 3 —uno en la ciudad de Qiqihar, provincia de Heilongjiang, China, y el otro en la ciudad de Yinchuan, Región Autónoma Hui de Ningxia, China— como sujetos del estudio, mediante un muestreo por conveniencia entre julio de 2024 y julio de 2025. Los criterios de inclusión fueron los siguientes: 1) edad ≥18 años; 2) un diagnóstico que cumpla con los criterios más recientes de la Organización Mundial de la Salud (OMS) para la leucemia aguda, confirmado mediante punción de médula ósea; y 3) comprensión del estudio y participación voluntaria en el mismo. Los criterios de exclusión fueron los siguientes: (1) antecedentes de otros tumores malignos o de deterioro grave de órganos vitales (p. ej., hígado, riñones o pulmones); (2) deficiencias cognitivas o de comunicación que impidan completar el cuestionario; y (3) antecedentes de trastornos psiquiátricos.

Para la investigación cuantitativa, el tamaño de la muestra debe ser al menos de cinco a diez veces el número de variables del estudio^(13)^. Este estudio incluyó 24 variables independientes. Teniendo en cuenta factores como las respuestas inválidas al cuestionario, el tamaño de la muestra para la encuesta se incrementó en un 20 %, lo que dio como resultado un rango de 150 a 300 casos. Este estudio incluyó un total de 289 casos.

En este estudio cualitativo, se empleó un muestreo intencional con variación máxima para reclutar a pacientes con LA como sujetos de investigación que representaran diversos géneros, edades, niveles educativos y otras características. Se realizaron entrevistas semiestructuradas. El tamaño de la muestra se determinó de acuerdo con el principio de saturación de la información. La recolección de datos se detuvo después de realizar dos entrevistas adicionales sin que surgieran nuevos temas. Finalmente, el análisis incluyó a 10 pacientes.

### MEDIDAS

#### Características Sociodemográficas

El investigador diseñó el cuestionario de la encuesta basándose en estudios previos y marcos teóricos. En él se solicitaba información demográfica, como el género, la edad y el nivel educativo, así como detalles relacionados con la enfermedad, como la duración de la enfermedad, la duración de la hospitalización y la presencia de afecciones crónicas.

#### Escala de Preparación para el Alta Hospitalaria (Readiness for Hospital Discharge Scale - RHDS)

Este estudio utilizó la escala adaptada al chino por Lin et al.^(14)^ en 2014. La escala cuenta con tres dimensiones y 12 ítems: Estado personal (ítems 1–3), Capacidad de adaptación (ítems 4–8) y Apoyo anticipatorio (ítems 9–12). La puntuación total varía de 0 a 120, y cada ítem se califica de 0 a 10. Las puntuaciones de 9 a 10 indican un nivel muy alto de preparación, de 8 a 8,9 un nivel alto, de 7 a 7,9 un nivel moderado y de 0 a 7 un nivel bajo. Una puntuación total más alta indica una mayor preparación para el alta. El coeficiente α de Cronbach para esta escala es de 0,890. En este estudio, es de 0,893.

#### Escala de Calidad de la Instrucción Para el Alta (Quality of Discharge Teaching Scale - QDTS)

La versión china de la Escala de Calidad de la Instrucción para el Alta fue revisada por el académico chino Wang et al.^(15)^. La escala, desarrollada por Weiss and Lokken^(16)^, comprende tres dimensiones: el contenido que se debe dominar antes del alta, el contenido que se dominó realmente antes del alta y la efectividad de las habilidades de instrucción para el alta. La escala consta de 24 ítems en total. Mide la calidad de las instrucciones de alta calculando las puntuaciones totales de las dimensiones “conocimientos reales adquiridos antes del alta” y “habilidades y efectividad de las instrucciones de alta”. Las puntuaciones totales más altas indican una mejor calidad de las instrucciones. El coeficiente α de Cronbach para esta escala es de 0,920. En este estudio, es de 0,939.

#### Escala de Evaluación del Apoyo Social (Social Support Rating Scale - SSRS)

Xiao^(17)^ desarrolló esta escala de 10 ítems en 1987. Comprende tres dimensiones: apoyo objetivo, apoyo subjetivo y utilización del apoyo. Los criterios de puntuación son los siguientes: los ítems 1 a 4 y 8 a 10 permiten solo una selección por ítem y se puntúan de 1 a 4 puntos, respectivamente. El ítem 5 se subdivide en cuatro categorías (A, B, C y D), cada una de las cuales se califica de 1 a 4 puntos, que van desde “ninguno” hasta “apoyo total”. Los ítems 6 y 7 se califican de la siguiente manera: 0 puntos si se selecciona “sin fuentes” y el número de fuentes enumeradas en “las siguientes fuentes” se cuenta como puntos. La puntuación total de la SSRS es la suma de las puntuaciones de los diez ítems. El coeficiente α de Cronbach para esta escala es de 0,892. En este estudio, es de 0,838.

#### Escala Hospitalaria de Ansiedad y Depresión (hospital Anxiety and Depression Scale - HADS)

Fue desarrollada por Zigmond and Snaith^(18)^ en 1983. Esta escala detecta posibles síntomas de ansiedad y depresión en pacientes hospitalizados. Cuenta con dos dimensiones, ansiedad y depresión, y un total de 14 ítems. Las puntuaciones de 0 a 7 en cualquiera de las dimensiones indican la ausencia de síntomas. Las puntuaciones de 8 a 10 sugieren la presencia de posibles síntomas, mientras que las puntuaciones de 11 a 21 los confirman. El coeficiente α de Cronbach para esta escala es de 0,875. En este estudio, es de 0,927.

#### Esquema de la Entrevista

Con base en una revisión bibliográfica y en discusiones del equipo, el investigador desarrolló un esquema preliminar de entrevista enfocado en los factores que promueven u obstaculizan la preparación para el alta entre los pacientes con LA. El esquema se basó en la teoría de la transición. Seis expertos en hematología y oncología revisaron el esquema, y dos pacientes con LA que cumplían con los criterios participaron en entrevistas piloto para finalizar el marco.

#### Recolección de Datos

La jefa de enfermería o la enfermera asignada al paciente se encargó de la recolección de datos cuantitativos. Estos explicaron a los pacientes el propósito y la importancia del estudio y obtuvieron su consentimiento antes de la recolección de datos. Los investigadores realizaron entrevistas individuales en el lugar utilizando preguntas estandarizadas. Para los datos cualitativos, investigadores capacitados en técnicas de entrevista cualitativa llevaron a cabo entrevistas grabadas en su totalidad, centrándose en documentar las señales no verbales de los pacientes, como expresiones faciales y gestos. Dentro de las 24 horas posteriores a la entrevista, el primer y el segundo autor transcribieron las grabaciones de audio y las notas escritas a mano al texto. Cualquier contenido poco claro se aclaró mediante un seguimiento telefónico con los pacientes.

#### Análisis de Datos

Se utilizó el software SPSS 26,0 para el análisis de datos cuantitativos. Las comparaciones entre grupos se realizaron mediante pruebas t o ANOVA unidireccional, y las correlaciones se evaluaron utilizando el método de Pearson. Se llevó a cabo un análisis de regresión lineal multivariante con la puntuación total del RHDS como variable dependiente. Para garantizar la solidez, los modelos se sometieron a pruebas de colinealidad (factor de inflación de la varianza [Variance Inflation Factor - VIF]), independencia de los residuos (Durbin–Watson) y normalidad. El análisis de los datos cualitativos se llevó a cabo utilizando el método de siete pasos de Colaizzi^(19)^. Dos investigadores codificaron y verificaron los datos de manera independiente, y las discrepancias se resolvieron mediante discusión grupal. Se empleó la verificación por parte de los participantes para garantizar la confiabilidad de los resultados.

### Consideraciones Éticas

Este estudio fue aprobado por el comité de ética del hospital (n.º de aprobación 2024-02-005-01). Todos los participantes dieron su consentimiento informado y se inscribieron voluntariamente en el estudio. Investigadores capacitados llevaron a cabo la recolección de datos cualitativos. Las entrevistas se grabaron en audio y se transcribieron. La verificación por parte de los participantes y los controles de confiabilidad entre evaluadores aseguraron la credibilidad y la consistencia de los datos. El estudio se llevó a cabo de acuerdo con la Declaración de Helsinki.

## RESULTADOS

### Características de la Muestra

Tras la recolección de los datos, dos investigadores revisaron y verificaron los cuestionarios de manera independiente. Con base en criterios de control de calidad predefinidos, como la presencia de errores lógicos evidentes o respuestas repetitivas, se excluyeron dos cuestionarios inválidos. Finalmente, el análisis incluyó 289 cuestionarios válidos, lo que arrojó una tasa de respuesta del 99,3 %. La mayoría de los pacientes eran mujeres (58,1 %), tenían 60 años o más (44,3 %), contaban con un nivel educativo de secundaria o inferior (61,9 %) y procedían de zonas rurales (48,1 %). Las características generales de los pacientes se resumen en la Tabla 1.

**Tabla 1:** Resultados del análisis univariante de la información general y la preparación para el alta de los participantes en el estudio (n = 289) – Heilongjiang, China, 2025.

Variables	Número de participantes (porcentaje)	Preparación para el alta (escore, X± S)	t/F	P-valor
Género			3,753^1)^	<0,001
Hombre	121(41,9)	97,21 ± 14,57		
Mujer	168(58,1)	89,43 ± 19,15		
Edad			5,238^2)^	0,006
<45	68(23,5)	97,38 ± 17,21		
45–60	93(32,2)	94,03 ± 18,92		
≥60	128(44,3)	89,21 ± 16,59		
Nivel educativo			17,542^2)^	<0,001
Secundaria o menor	179(61,9)	88,11 ± 17,83		
Preparatoria o universidad	93(32,2)	99,77 ± 15,08		
Licenciatura o superior	17(5,9)	102,12 ± 14,89		
Estado civil			4,253^1)^	<0,001
Casado	227(78,5)	94,94 ± 16,24		
No casado/Divorciado/Viudo	62(21,5)	84,42 ± 20,62		
Lugar de residencia			20,385^2)^	<0,001
Rural	139(48,1)	86,27 ± 18,18		
Pueblo	43(14,9)	101,00 ± 14,55		
Urbano	107(37,0)	97,67 ± 15,40		
Vive con su familia			2,676^1)^	0,008
Sí	273(94,5)	93,36 ± 17,62		
No	16(5,5)	81,25 ± 17,03		
Método de pago de gastos médicos			10,786^2)^	<0,001
Nuevo Plan de Salud Cooperativo Rural	126(43,6)	86,30 ± 17,58		
Seguro médico para residentes urbanos	83(28,7)	97,80 ± 16,86		
Seguro médico para empleados	74(25,6)	97,03 ± 16,22		
Otro	6(2,1)	102,50 ± 10,46		
Ingresos mensuales per cápita del hogar			36,022^2)^	<0,001
<3000	162(56,1)	86,10 ± 18,89		
3000–5000	95(32,9)	98,61 ± 11,28		
>5000	32(11,1)	108,44 ± 10,23		
Cuidador principal			10,298^2)^	<0,001
Padres	27(9,3)	100,04 ± 17,35		
Cónyuge	141(48,8)	96,81 ± 14,18		
Hijos	100(34,6)	86,39 ± 20,27		
Otro	21(7,3)	85,52 ± 16,48		
Situación laboral			13,152^2)^	<0,001
Desempleado	177(61,2)	89,52 ± 18,47		
Renunció/Jubilado	85(29,4)	94,73 ± 15,43		
Activo	27(9,3)	107,00 ± 11,27		
Duración de la enfermedad			16,829^2)^	<0,001
<1 mes	29(10,0)	80,21 ± 16,96		
1–6 meses	49(17,0)	85,39 ± 13,79		
>6 meses	211(73,0)	96,09 ± 17,49		
Comorbilidad con enfermedades crónicas			–6,601^1)^	<0,001
Sí	130(45,0)	85,56 ± 18,73		
No	159(55,0)	98,51 ± 14,61		
Duración de la estadía			2,373^2)^	0,070
<7 días	71(24,6)	92,89 ± 19,02		
7–14 días	113(39,1)	94,03 ± 16,83		
14–21 días	58(20,1)	87,43 ± 15,02		
≥21 días	47(16,3)	95,64 ± 20,23		
Nivel de conocimiento sobre la enfermedad			55,303^2)^	<0,001
Muy familiarizado	163(56,4)	101,56 ± 13,58		
Algo familiarizado	72(24,9)	85,69 ± 15,24		
No muy familiarizado	40(13,8)	77,28 ± 15,88		
Para nada familiarizado	14(4,8)	69,36 ± 9,94		
Reingreso no programado en 30 días			–6,262^1)^	<0,001
Si	35 (12,1)	76,11 ± 16,78		
No	254(87,9)	94,97 ± 16,69		
Disposición para recibir alta			10,036^1)^	<0,001
Si	249(86,2)	96,31 ± 15,12		
No	40(13,8)	70,13 ± 16,50		

Nota:*t*= prueba*t*para muestras independientes; *F*= ANOVA unidireccional;

^1)^ = valor de t;

^2)^ = valor de F.

### Las Puntuaciones del RHDS de los Pacientes con LA

La puntuación en la escala RHDS de los pacientes con LA fue de 92,69 ± 17,77, con sub-puntuaciones de 21,96 ± 6,13 para la situación personal, 38,09 ± 10,04 para la capacidad de adaptación y 32,63 ± 6,83 para el apoyo anticipado. La puntuación media para todos los ítems fue de 7,72 ± 1,48, lo que indica un nivel moderado de preparación para el alta.

#### Análisis Univariante (Tabla 1 Y Tabla 2)

**Tabla 2:** Análisis de correlación de la preparación para el alta entre los participantes del estudio (N = 289) – Heilongjiang, China, 2025.

Variable	Preparación para el alta	Calidad de las instrucciones al alta	Apoyo social	Ansiedad y depresión
Preparación para el alta	1			
Calidad de las instrucciones para el alta	0,737**	1		
Apoyo social	0,570**	0,542**	1	
Ansiedad y depresión	–0,597**	–0,695**	–0,502**	1

Nota: **P < 0,01.

### Análisis de RegresiÓn Lineal Multivariante

Se realizó un análisis de regresión lineal multivariante utilizando el puntaje total del RHDS como variable dependiente y las variables estadísticamente significativas del análisis univariante como variables independientes. Los resultados indican que el nivel educativo, el lugar de residencia, el método de pago de los servicios médicos, la presencia de enfermedades crónicas comórbidas, el nivel de conocimiento sobre la enfermedad, la ocurrencia de reingresos no planificados en un plazo de 30 días, la preparación para el alta, la calidad de las instrucciones de alta y el apoyo social influyen en la preparación para el alta entre los pacientes con LA. Estos factores explican el 71,7 % de la varianza total (Tabla 3).

**Tabla 3:** Análisis de regresión lineal multivariante de la preparación para el alta entre los participantes del estudio (N = 289) – Heilongjiang, China, 2025.

Variables	B	SE	β	*t*	P
Constante	29,999	11,679		2,569	0,011
Nivel educativo	3,173	1,280	0,108	2,479	0,014
Residencia = Ciudad	8,395	2,297	0,168	3,655	<0,001
Residencia = Pueblo	7,378	2,802	0,201	2,634	0,009
Forma de pago de los servicios médicos = Seguro médico para residentes urbanos	5,929	2,044	0,151	2,901	0,004
Seguro médico para empleados	13,729	2,909	0,338	4,719	<0,001
Comorbilidad de enfermedades crónicas	4,093	1,300	0,115	3,149	0,002
Nivel de conocimiento sobre la enfermedad	–2,171	1,021	–0,109	–2,128	0,034
Si hubo un reingreso no planificado en los 30 días siguientes	6,693	1,893	0,123	3,536	<0,001
Preparación para el alta	–7,172	1,988	–0,140	–3,607	<0,001
Calidad de las instrucciones al alta	0,310	0,046	0,369	6,682	<0,001
Apoyo social	0,257	0,096	0,130	2,661	0,008

Nota: R^2^ = 0,742, R^2^ ajustado = 0,717; *F* = 30,191, *P* < 0,001.

### Hallazgos de la Investigación Cualitativa

#### Tema 1: Impacto de las Circunstancias Personales en la Preparación Para el Alta

La preparación de un paciente para el alta está estrechamente relacionada con su funcionamiento físico, sus habilidades psicológicas para afrontar la situación y su comprensión de la enfermedad. Debido a los efectos secundarios de la quimioterapia, la mayoría de los pacientes experimentan una disminución de la fuerza física y malestar por los síntomas, lo que afecta aún más su estado psicológico. Algunos pacientes desarrollan gradualmente patrones cognitivos positivos durante una enfermedad prolongada, lo que mejora su capacidad para afrontar la situación.

*“Después de la quimioterapia, me siento extremadamente fatigado. En casa, solo puedo descansar; cualquier actividad empeora el malestar” (P3).*

*“A pesar de la presión económica, poco a poco he aprendido a aceptarla, viéndola como un sacrificio para un futuro mejor” (P2).*

#### Tema 2: Papel de los Sistemas de Apoyo Externos

El apoyo familiar desempeña un papel fundamental en la recuperación del paciente, ya que alivia la ansiedad y aumenta la confianza. Por otro lado, la ayuda económica y el aliento emocional por parte de la sociedad y el gobierno pueden reducir significativamente las cargas de los pacientes.

*“Mi familia me cuida todos los días, lo que me hace sentir increíblemente seguro.” (P6).*

*“El gobierno me proporcionó asistencia para personas de bajos ingresos, lo que alivió considerablemente mi carga económica.” (P8).*

#### Tema 3: Papel de apoyo de las Intervenciones de Enfermería

Las instrucciones estandarizadas al dar de alta y la continuidad de los servicios de atención mejoran la capacidad de los pacientes para manejar su enfermedad y brindan apoyo continuo para la atención en el hogar. Los pacientes mencionaron de manera constante que la orientación de los profesionales de la salud reforzó su confianza en el autocontrol.

*“La enfermera me explicó en detalle cómo manejar mi cuidado en casa, lo que me dio mayor tranquilidad.” (P4).*

*“Después del alta, los médicos y las enfermeras me dieron seguimiento a través de WeChat, respondiendo de inmediato a cualquier pregunta que tuviera.” (P2).*

#### Tema 4: Percepción y TransformaciÓn del rol

Durante la transición de la enfermedad a la salud, los pacientes pasaron de la ansiedad y la impotencia a una aceptación y adaptación graduales. Una percepción positiva del rol mejoró la preparación para el alta y la reintegración social.

*“Al principio, me sentía limitado por mi enfermedad. Ahora que me han dado de alta, no me siento diferente a los demás” (P5).*

#### Hallazgos de métodos mixtos

Enmarcado en la teoría de la transición, este estudio reveló que la preparación de los pacientes con LA para el alta muestra complementariedad en el nivel de la condición de transición y extensibilidad en los niveles de la intervención de enfermería y la naturaleza de la transición.

## DISCUSIÓN

### Enfocarse en las Circunstancias Individuales de los Pacientes, Comprender sus Necesidades y Guíarlos Hacia Estrategias Proactivas de Afrontamiento

En cuanto a la situación individual, la investigación cuantitativa y la cualitativa se complementan entre sí. La investigación cuantitativa, respaldada por datos, identificó los niveles de educación y de conocimiento sobre la enfermedad como dos variables clave. Estos hallazgos se validan en diversas enfermedades^(20)^. La investigación cualitativa profundiza en las causas subyacentes de la situación individual, revelando el concepto de competencia en el autocuidado y complementando los hallazgos cuantitativos. Los resultados sugieren que los pacientes con sólidas capacidades de autocuidado prestan mucha atención a los detalles del control de infecciones y pueden adaptarse según sus circunstancias. Esta capacidad de autocuidado establece un marco claro antes del alta. Por el contrario, los pacientes con capacidades de autocuidado más débiles mostraron confusión y ansiedad. Un paciente mencionó esto en una entrevista: “No sé qué hacer una vez que llegue a casa”. Su falta de una planificación adecuada del autocuidado disminuyó la eficacia de la preparación para el alta. Este hallazgo concuerda con los resultados cuantitativos, los cuales sugieren que las personas con un mayor nivel educativo suelen tener mayores habilidades de aprendizaje para el autocuidado. De acuerdo con la teoría Conocimiento-Creencia-Comportamiento (Knowledge - Beliefs - Behavior), cuanto mayor es la comprensión que tiene una persona sobre el tratamiento de la enfermedad, mayor es su motivación para el autocuidado^(21)^. Los dos estudios se complementan entre sí. El estudio cuantitativo estableció un marco para evaluar el estado individual e identificó los factores que influyen en la preparación de los pacientes con EA para el alta. El estudio cualitativo explicó los fundamentos y los mecanismos subyacentes a través de los cuales estas características ejercen su influencia. Por lo tanto, se recomienda que los profesionales de la atención de salud evalúen de manera proactiva el estado físico y mental de los pacientes, así como sus capacidades de autocuidado. Deben alentar activamente a los pacientes a transformar el cumplimiento médico pasivo en una participación activa en la rehabilitación. Al mismo tiempo, deben desarrollar herramientas educativas visuales e interactivas adaptadas a los pacientes con bajos niveles de educación para reducir la complejidad de la educación para la salud y mejorar de manera efectiva la preparación para el alta.

### Creación y Mejora de los Sistemas de Apoyo al Paciente

La investigación cuantitativa indica que el apoyo social de los pacientes influye en su preparación para el alta. Este hallazgo concuerda con el tema de los “sistemas de apoyo externos” identificado en la investigación cualitativa. Las declaraciones de los pacientes en las entrevistas, tales como “Mi cónyuge renunció a su trabajo para brindarme cuidados a tiempo completo” y “Cuidado integral por parte de los familiares”, corroboran el consuelo emocional y el cuidado familiar, los cuales son formas de apoyo subjetivo. Por el contrario, ejemplos como “financiamiento de Waterdrop” y “reembolso a través de subsidios de subsistencia del gobierno” demuestran el papel práctico de la asistencia material dentro del apoyo objetivo. Además, la investigación cuantitativa reveló que los pacientes que residen en zonas rurales están menos preparados para el alta que aquellos que viven en zonas urbanas. Un análisis en profundidad sugiere que los pacientes urbanos pueden acceder al tratamiento médico más rápidamente en casos de emergencia. Esto se traduce en menor ansiedad y una mayor sensación de seguridad, lo que mejora su preparación para el alta^(22)^. Por lo tanto, se recomienda que los profesionales de la salud reconozcan el importante papel de los cuidadores y alienten a los familiares a desempeñar un papel activo en el cuidado de los pacientes. Esto brindará a los pacientes un mayor apoyo. Al mismo tiempo, se deben fortalecer los esfuerzos de colaboración entre los hospitales, las comunidades y las familias. Se deben establecer centros de continuidad de la atención en colaboración con instituciones de atención primaria para realizar seguimientos regulares y ofrecer consultas de telemedicina. Se deben mejorar las redes de apoyo social y promover la integración de los seguros médicos y los programas de beneficencia. En particular, se debe establecer un sistema de asistencia específico para atender las necesidades médicas y aliviar las cargas financieras de las poblaciones rurales.

### Mejorar la Comunicación Entre Enfermeras y Pacientes y la Continuidad de los Servicios de Atención

Los resultados sugieren que la calidad de las instrucciones de alta influye en la preparación para el alta, lo cual concuerda con los hallazgos de estudios previos^(23,24)^. La investigación cualitativa también indica que la continuidad de los servicios de atención puede mejorar la preparación de los pacientes para el alta. El análisis indica que esto puede deberse a que los pacientes con leucemia linfoblástica aguda (LLA) no se han recuperado por completo al momento de recibir la alta. Las reacciones tóxicas relacionadas con el tratamiento coexisten con necesidades de autocuidado a largo plazo, lo que genera una mayor demanda de orientación en salud en comparación con la población general de pacientes. La continuidad precisa y oportuna de los servicios de atención de salud puede aumentar de manera efectiva la confianza de los pacientes para regresar a casa y su capacidad para manejar su propio cuidado. Esto reduce la incidencia de complicaciones y mejora la preparación para el alta. Por lo tanto, se recomienda que los profesionales de la salud optimicen la orientación para la preparación del alta y involucren a los pacientes a través de diversas iniciativas educativas. Al mismo tiempo, se debe mejorar la calidad de los servicios de continuidad de la atención. Los hospitales podrían establecer plataformas para brindar servicios de atención continua mediante llamadas telefónicas regulares, WeChat y métodos de “Internet + Enfermería”^(25)^. Estas medidas abordarían las inquietudes de los pacientes después de salir del hospital y proporcionarían un apoyo integral de rehabilitación durante todo el ciclo.

### Enfoque en la Transición de la Enfermedad y la Reconstrucción del rol Social

Los resultados del estudio indican que los pacientes con enfermedades crónicas o reingresos no planificados están menos preparados para el alta, lo cual concuerda con los hallazgos de Yang et al.^(26)^. Estos pacientes requieren planes de tratamiento y atención más complejos, lo que puede conducir a un aumento significativo de las complicaciones y los reingresos no planificados. El análisis cualitativo reveló que la percepción del rol y la transición son temas clave que influyen en la preparación para el alta. Desde la perspectiva de la teoría de la transición, los pacientes de LA experimentan una transición ambiental durante la fase de alta, así como un proceso de reconstrucción de la identidad —específicamente, un cambio de ser pacientes hospitalarios dependientes a convertirse en cuidadores principales responsables del autocuidado en el hogar. Este proceso de transición de identidad implica ajustes en el autoconcepto. Según la teoría de la autorregulación^(27)^, cuando las personas se enfrentan a una amenaza significativa para su salud, desarrollan estrategias de conducta relevantes basadas en su comprensión de la enfermedad. Los pacientes que perciben su enfermedad como una afección crónica manejable son más propensos a adoptar comportamientos proactivos de afrontamiento, mejorando así su preparación para el alta. Por su parte, la teoría de la identidad de la enfermedad sugiere que los pacientes con enfermedades crónicas deben integrar su identidad de enfermedad en su autoconcepto^(28)^. En este estudio, algunos pacientes demostraron una integración activa de la identidad. Por ejemplo, dijeron: “Me habré recuperado por completo una vez que me den de alta”, lo que refleja la reconstrucción de su identidad de enfermedad. Por lo tanto, prepararse para el alta implica más que adquirir conocimientos; también implica reconstruir la propia identidad y pasar por una transición psicológica. Las intervenciones futuras deberían ir más allá de la educación básica en salud para incluir el empoderamiento en el rol y la integración de la identidad, con el fin de mejorar la capacidad de autogestión de los pacientes.

## CONCLUSIÓN

Este estudio utilizó un diseño convergente de métodos mixtos y halló que los pacientes con leucemia aguda presentan niveles moderados de preparación para el alta. Este hallazgo confirma que la preparación se ve influida por una interacción multidimensional de factores, entre los que se incluyen las características personales, el apoyo externo, los servicios de enfermería y las percepciones de los roles de salud. Sin embargo, los modelos de enfermería existentes presentan deficiencias en el manejo a largo plazo desde el hospital hasta el hogar. Estos modelos priorizan el control de los síntomas, mientras descuidan el desarrollo sostenible de la adaptación psicosocial y las capacidades de autogestión.

El estudio presentó varias limitaciones. En primer lugar, la muestra se restringió a dos hospitales terciarios de grado A en el norte de China, lo que podría limitar la generalizabilidad de los resultados. En segundo lugar, existieron desafíos metodológicos al brindar educación en salud a pacientes con bajo nivel de alfabetización. En tercer lugar, la continuidad de los servicios de atención de salud es limitada en las zonas rurales, y las deficiencias estructurales pueden afectar los resultados de la rehabilitación a largo plazo. Las investigaciones futuras podrían enfocarse en lo siguiente: 1. Realizar estudios multicéntricos, con muestras amplias y de carácter longitudinal para dar seguimiento dinámico a los cambios en la preparación para el alta y sus factores influyentes, así como para identificar los momentos críticos de intervención. 2. Desarrollar y validar herramientas de educación para la salud visuales e interactivas para aumentar la concientización sobre la enfermedad y las capacidades de autogestión entre los pacientes con bajos niveles de alfabetización. 3. Establecer un modelo digital de continuidad de la atención basado en la colaboración entre “hospital, comunidad y hogar”. Este modelo debería prestar especial atención a la accesibilidad de los servicios para los pacientes rurales, optimizar la asignación de recursos de enfermería y mejorar la calidad de vida a largo plazo. La integración de los hallazgos de la investigación con los marcos teóricos proporciona estrategias prácticas para las intervenciones relacionadas con la preparación para el alta, haciendo hincapié en el papel fundamental de los mecanismos interactivos multidimensionales para mejorar los resultados de rehabilitación y la calidad de vida de los pacientes con leucemia aguda.

## Data Availability

El conjunto de datos que respaldan las conclusiones de este estudio está disponible previa solicitud al autor correspondiente.
